# Jail-based opioid treatment implementation, outcomes, and costs: lessons learned from the Massachusetts Justice Community Opioid Innovation Network (MassJCOIN)

**DOI:** 10.1186/s12954-026-01566-z

**Published:** 2026-10-23

**Authors:** Elizabeth A. Evans, Thomas J. Stopka, Ekaterina Pivovarova, Danielle Ryan, Sean M. Murphy, Kaitlyn Jaffe, Dana Bernson, Benjamin Bovell-Ammon, Kathryn E. McCollister, Elyse Bianchet, Randall Hoskinson, Peter D. Friedmann

**Affiliations:** 1 Department of Health Promotion and Policy, School of Public Health and Health Sciences, University of Massachusetts Amherst, 312 Arnold House, 715 North Pleasant Street, Amherst, MA 01003 USA; 2 Department of Public Health and Community Medicine, Tufts University School of Medicine, 136 Harrison Avenue, Boston, MA 02111 USA; 3 Department of Psychiatry, University of Massachusetts Medical School and Massachusetts Center of Excellence for Specialty Courts, Worcester, MA USA; 4 Department of Family Medicine and Community Health, University of Massachusetts Medical School, 55 Lake Avenue North, Worcester, MA 01655 USA; 5 Department of Population Health Sciences, Weill Cornell Medical College, 575 Lexington Ave, New York, NY USA; 6 Office of Health Data, Strategy, and Innovation, Massachusetts Department of Public Health, 250 Washington Street, Boston, MA 02108 USA; 7 UMass Chan Medical School–Baystate and Baystate Medical Center, Springfield, MA USA; 8 Soffer Clinical Research Center, Department of Public Health Sciences, University of Miami Miller School of Medicine, 1120 NW 14th Street, Suite 1019, Miami, FL 33136 USA; 9 Department of Population & Quantitative Health Science, University of Massachusetts Chan Medical School, Worcester, MA 01655 USA

**Keywords:** Opioid use disorder, Massachusetts justice community opioid innovation network (MassJCOIN), Carceral-legal settings, Buprenorphine, Methadone, Naltrexone, Medications for opioid use disorder (MOUD)

## Abstract

Limited access to medications for opioid use disorder (MOUD) is a major driver of the opioid overdose crisis. Jails have been slow to implement MOUD treatment, especially agonist medications (methadone, buprenorphine). Massachusetts mandated certain county jails pilot-test delivery of all FDA-approved MOUD treatments, stipulating jails continue MOUD, or initiate it prior to release, and facilitate MOUD continuation in the community post-release. The Massachusetts Justice Community Opioid Innovation Network (MassJCOIN) partnered with seven pilot jails, the Massachusetts Department of Public Health, and community treatment providers to conduct a Type 1 hybrid effectiveness-implementation study of this carceral MOUD program. Drawing from selected MassJCOIN evidence on MOUD treatment program implementation, outcomes, and costs, we consider how findings can inform more effective, humane public health and clinical responses to treating incarcerated individuals. MOUD treatment in jail improves treatment engagement, reduces recidivism, and saves lives. MOUD program implementation facilitators included: policies and processes to offer *universal MOUD access*; *transformative and adaptive leadership*; *appropriate staffing capacity and capabilities*; proactive *prevention of MOUD diversion*; and consideration of *patient preferences* for MOUD treatment and *co-occurring mental health conditions*. MOUD program success was influenced by the *legislative mandate* and other major statewide changes and a *community-based treatment system* conducive to MOUD implementation. MOUD treatment continuity post-release was facilitated by *re-entry planning* and preparing individuals for *post-release risk environments*. Site-specific costs of implementing and sustaining MOUD programming provide information for decisions about program design, budgetary needs, and resource allocation. Carceral MOUD programming is a compelling strategy to advance public safety and public health. Lessons learned provide a playbook for broader diffusion of carceral MOUD programs and can help carceral leadership, healthcare providers, and policymakers make carceral healthcare improvements. Within a harm reduction framework, carceral MOUD treatment can provide substantial opportunities to fill gaps in public health prevention efforts and clinical care to reduce opioid overdose and other concomitant risks. Carceral MOUD programming offers ways for carceral systems to work for the common good, garner trust in public institutions, and increase their legitimacy and value as public institutions.

## Introduction

Limited access to effective medications for opioid use disorder (MOUD) that reduce opioid overdose risks is a major driver of the overdose crisis. Jails have been slow to implement MOUD treatment options, especially the opioid agonist medications methadone and buprenorphine, which places incarcerated people at high risk for opioid overdose upon their return to the community [1–3]. A 2018 law (Chap. 208 of the Acts of 2018) made Massachusetts the first state to mandate that certain county jails deliver all FDA-approved MOUD treatment options (buprenorphine, methadone, naltrexone). Chapter 208 established a 4-year pilot program to expand MOUD access at five jails, with two more jails voluntarily joining. The law stipulated that jails continue MOUD for individuals receiving it prior to detention and initiate it prior to release among sentenced individuals with opioid use disorder where appropriate. Jails were also required to facilitate continuation of MOUD in the community upon release. In effect, implementation of the MOUD program in these jail settings occurred within a setting that included statutory requirements, some financial support, state agency involvement, the involvement of community-based treatment partners, and broader statewide support toward MOUD access.

The Massachusetts Justice Community Opioid Innovation Network (MassJCOIN) partnered with these seven jails, with diverse populations and models across urban, suburban, and rural counties, as well as the Massachusetts Department of Public Health and community treatment providers to conduct a Type 1 hybrid effectiveness-implementation study of Chap. 208 [4]. Since 2019, we have assessed the implementation, outcomes, and costs of this pilot program.

The objective of this commentary is to consider how selected findings from MassJCOIN publications can inform more effective, humane public health and clinical responses to treating incarcerated individuals. MassJCOIN is one of more than 30 clinical research hubs within the JCOIN network, a federally funded initiative designed to advance scientific knowledge on effective policies, practices, and interventions, and to expand their quotidian use within health and justice settings [5]. This commentary highlights evidence accumulated over seven years of study (Table 1). We focus on MOUD treatment program implementation, outcomes, and costs to inform policy, broaden the health impacts, and reduce opioid-related overdose and concomitant risks as other states and jurisdictions begin to offer MOUD treatment in carceral settings.

**Table 1:** MassJCOIN evidence on implementation, outcomes, and costs of program to provide medications for opioid use disorder (MOUD) in county jails

Author	Year	Study type	Data source	EPIS	Main findings/implications	Principles & context
Evans et al. [6]	2022	1	Jail staff	1	With appropriate protocols, buprenorphine diversion within jail MOUD programs is perceived to be uncommon and preventable by jail staff. Promising practices in program design help limit diversion and inform correctional officials and lawmakers as they consider if and how to provide MOUD treatment in carceral settings.	Stigma reduction; Increasing access to evidence-based care
Evans et al. [7]	2023	1	Patients	1	Formerly incarcerated patients perceived MOUD diversion within jail programs as occurring less often than expected and able to be reduced with appropriate protocols. To help limit diversion, patients recommended provision of MOUD upon intake to all individuals with opioid use disorder who need it.	Evidence-based, humane healthcare; Low barrier care; Therapeutic approaches
Santelices et al. [8]	2025	1	Patients	1	Participants stressed that MOUD programs should be patient-centered and guided by patients’ symptoms and needs, rather than by policies that postpone or limit care.	Patient-centered care, humane treatment; Therapeutic approach
Santelices et al. [unpublished observations]	2026	1	County sheriffs	1	Implementation of jail-based MOUD programs depends on sheriffs’ transformative and adaptive leadership capacity. Successful implementation strategies relied on promoting staff engagement and buy-in, navigating funding and policy, and strengthening interagency collaboration to secure reentry resources and continuity of care post release.	Stigma reduction; Build institutional capacity for evidence-based, humane care; Treatment continuity
Pivovarova et al. [9]	2022	1	Jail staff	1, 2	Implementation of MOUD in jails requires flexibility, and collaboration with and reliance on external agencies. Leaders and policymakers must recognize the cultural shift inherent in such programs and allow for resources and education to assure program success.	Stigma reduction; Build institutional capacity for evidence-based, humane care
Michener et al. [10]	2024	3	Jail staff	1	Findings underscore the importance of organizational climate for successful implementation of jail MOUD programs and provide support for medication-specific educational resources to improve jail staff perceptions of MOUD.	Stigma reduction; Build institutional capacity for evidence-based, humane care
Stopka et al. [12]	2024	1	Patients	1	Understanding patient preferences can inform protocols in jails considering implementation of long-acting injectable buprenorphine treatment. If used in response to medication diversion, efforts should be made to include patients in decision-making.	Patient-centered, humane care
Pivovarova et al. [13]	2025	1	Jail staff	1	MOUD implementation in jails was perceived to reduce acute mental health crises and the demand for mental health services at jail intake. As jails work to meet MOUD regulatory requirements, leadership will need to manage potential staffing shortages, infrastructure constraints, and shifts in counseling services. Guidelines should consider unintended consequences of MOUD on other behavioral health services.	Holistic, patient-centered care; Humane treatment
Pivovarova et al. [16]	2025	1	MOUD provider staff	2	Community-based MOUD providers identified agency staffing, culture, and adaptability, community support, intentional relationship-building, and effective communication as essential for jail partnerships. Establishing key jail contacts, opportunities for cross-agency education and engagement, and support from other organizations can mitigate challenges posed by fragmented jail services and differing priorities.	Stigma reduction; Centering the dignity of people who use drugs
Matsumoto et al. [17]	2022	1	Jail staff	3	Post-release MOUD continuity of care requires training staff in reentry planning, and resources to enhance internal collaboration and partnerships between jail and community MOUD programs. Efforts to reduce systemic barriers related to unanticipated release timing and health insurance reinstatement are needed to optimize seamless post-release care.	Stigma reduction; Treatment continuity; Overdose risk reduction; Social determinants of health (SDOH) & structural barriers
Stopka et al. [18]	2022	1	MOUD provider staff	3	Community-based MOUD provider staff highlight the need for investments in care coordination, staffing, and funding to strengthen jail-to-community-based MOUD treatment, removing barriers to care continuity, and decreasing opioid overdose deaths during this high-risk transition.	Stigma reduction; Treatment continuity; Overdose risk reduction; SDOH
Stopka et al. [19]	2026	1	Patients	3	Patients recognized the high risk of MOUD treatment lapses during transitions from jail to community reentry, and valued MOUD continuity services initiated during incarceration and continued post-release. Variation in patient-reported quality of reentry planning services underscored the need for more standardized investment in re-entry and treatment continuity services.	Treatment continuity; Overdose risk reduction; Social determinants of health & structural barriers
Michener et al. [20]	2025	1	Patients	3	Patients identified physical, social, economic, and policy drivers of post-release overdose. Early MOUD induction during incarceration with continued treatment upon release, peer-driven harm reduction, and community reentry support were reported to be protective against overdose risk.	Evidence-based, humane care; Overdose reduction; SDOH & structural barriers
Friedmann et al. [21]	2025	2	Linked administrative data	All	Receipt of MOUD in jail was associated with an increased likelihood of post-release MOUD initiation and decreased risks of overdose, death from any cause, and reincarceration. MOUD implementation in carceral settings, potentially facilitated by opioid settlement funds and other policy initiatives, can reduce overdose, mortality, and reincarceration among people with OUD involved in the criminal- legal system.	Evidence-based, humane care; Overdose, mortality, and overall risk reduction
Ryan et al. [22]	2023	3	Jail staff	1, 2	The budget impact tool can help jail/prison leadership, policymakers, and other stakeholders identify/estimate the resources, costs, and relative effectiveness associated with various MOUD delivery models, and consider potential downstream cost offsets of successful treatment.	Impact policy to expand access to evidence-based, humane care
Ryan et al. [24]	2025	3	Jail staff	1, 2	Based on a cost analysis of three different models of jail MOUD program delivery, actionable insights for pre-implementation jails include engaging with similar jails that have implemented MOUD, building relationships with community-based MOUD treatment providers, and educating and training staff on OUD.	Impact policy to expand access to evidence-based, humane care

## Lessons learned on how to implement a carceral MOUD program

MassJCOIN conducted an implementation study to understand systemic and contextual factors that facilitate and impede delivery of MOUD in jail and community care coordination, as well as strategies that optimize delivery of these services. The conceptual basis for the study was that jails were engaged in the diffusion of an innovation—implementation and operation of a MOUD program—that involved multiple actors working collaboratively and with focused intention for the uptake and adaptation of new ideas, technologies, and practices. Guided by the Exploration, Preparation, Implementation, Sustainment (EPIS) framework, we collected comprehensive multi-wave mixed-methods data via jail staff surveys (*n* = 111) and in-depth qualitative interviews with jail staff (*n* = 245), current (*n* = 52) and formerly (*n* = 38) incarcerated individuals, and community MOUD treatment staff (*n* = 36). We highlight lessons learned for each of these EPIS domains: the inner jail context, the outer context or environment outside of jails, and bridging factors that influenced MOUD program adoption and success.

### Inner context of jails

*Universal MOUD access*. The law in Massachusetts stipulated that jails continue MOUD for individuals in jail who were already being treated with it in the community and that new MOUD inductions, where appropriate, occur at least 30 days prior to release. We documented variation by jail site in early implementation of MOUD programming, with few jails electing to conduct new MOUD inductions at jail entry or proactively outreaching to pre-trial individuals. We also documented that to increase the benefits of the MOUD program, patients and jail staff recommended universal provision of MOUD upon intake to all individuals with opioid use disorder [6–8]. A key takeaway from study results was that universal MOUD access would require re-organization of MOUD programming to identify all people with opioid use disorder who were not yet receiving MOUD at jail intake, inclusive of pre-trial and sentenced individuals, and provide MOUD to all eligible individuals who chose to be treated, with assessment and new inductions within 24-hours of jail entry.

*Transformative adaptive leadership*. Our team interviewed 11 county sheriffs to understand their experiences of implementing MOUD programs through transformative and adaptive leadership strategies [Santelices et al., unpublished observations]. When carceral institutional culture presented an obstacle to MOUD program implementation, sheriffs employed several approaches to counter negative perceptions of MOUD and mobilize staff (e.g., staff education and reallocation, building an in-house MOUD program to facilitate buy-in). Sheriffs also identified strong external partnerships and support as integral to the planning, launch, and sustainability of MOUD programs. Interagency collaborations, including peer jails, community providers, and state and national organizations, helped sheriffs to identify best practices, plan logistics and steps, and secure funding for program sustainability.

*Appropriate staffing capacity and capabilities*. Alongside leadership factors, clinical, correctional, and jail administrator participants described the critical nature of staffing practices and attitudes for successful implementation [9]. To offer MOUD services, jail staff took on additional responsibilities, such as supporting medication administration, data entry and reporting, and maintaining institutional safety amidst different treatment processes. Staff also contracted with community providers, whereas previously, most other health services were offered by jail medical and mental health staff. Jail staff participants described how jails hired additional correctional officers, MOUD clinicians, substance use counselors, and reentry navigators. Program implementation was facilitated by staff who were open to learning about and engaging in MOUD-related work, and had prior experience in working with individuals with substance use and mental health disorders. We learned that younger correctional officers and staff were more likely to be accepting of MOUD and not hold stigmatizing beliefs, partly because of lived experience with family and friends struggling with addiction. To improve existing attitudes among staff, particularly those who may have had erroneous beliefs and opposed introduction of MOUD, jails offered extensive education and training, emphasizing the importance of ongoing training. Findings were complemented by results from our jail staff survey, in which we learned how MOUD implementation could be facilitated by jails' organizational climate, including efforts to support positive staff attitudes toward organizational change and offering educational resources for staff to learn about specific medications [10].

*MOUD diversion*. Carceral officials often cite diversion of medication, particularly buprenorphine, as a reason for not offering MOUD treatment in jails and prisons. Contrary to these expectations, however, our results documented that jail staff and formerly incarcerated patients perceived MOUD diversion within carceral programs as occurring less often than expected and that it could be reduced with appropriate protocols [6, 7]. In part, the availability of prescribed MOUD reduced the incentive for diversion, because some of the demand for diverted MOUD was by individuals seeking to self-treat withdrawal symptoms. We identified best practices for jails to supervise delivery of MOUD and effectively reduce medication diversion [11]. Results also pointed to how staff and patients believed that MOUD diversion could be reduced by centering patients’ desire for a therapeutic treatment environment in carceral settings including, for example, use of treatment agreements, graduated consequences for actual and attempted diversion rather than immediate dose reductions, and carceral healthcare that is humanizing and promotes patient dignity and respect. Patient participants shared that it was useful for clinical staff to review a treatment agreement with patients prior to MOUD induction because it helped to clearly communicate expectations, the reasons for strict protocols during dosing, and the consequences for being involved in medication diversion or contraband activities [7]. A recommendation was for jails to focus on creating therapeutic treatment environments, for example by addressing medication diversion with collaborative deliberations that consider issues of safety, security, and health and by allowing clinical staff to work with patients to make informed treatment-related decisions [6, 7]. Optimal operation of a jail-based MOUD program requires use of evidence-based steps to proactively prevent MOUD diversion, and plans to address diversion as guided by therapeutic principles when it does occur.

*MOUD types and patient preferences*. The state mandated that jails offer all FDA-approved types of MOUD. To select the type of MOUD during incarceration, jail staff considered patient preferences, among other factors such as prior patient treatment history and medication availability in the facility the patient was expected to be transferred to or in the community of planned release. For example, participants shared concerns about how some patients who were treated with MOUD during incarceration in Massachusetts may be transferred to a carceral facility in another state that only offered naltrexone or did not offer any MOUD treatment. Another issue that shaped type of MOUD received during incarceration was whether the patient was returning to a community after release, such as in a rural setting, with limited access to certain types of MOUD. We identified reasons behind patient preferences for a particular MOUD, offering information that can be used to inform more effective and humane treatment. For example, we learned about patient participants’ experiences with and perspectives on extended-release buprenorphine, a relatively new treatment modality that can be injected weekly or monthly. Participants who viewed long-acting injectable buprenorphine (LAI-Bupe) favorably appreciated avoiding unpleasant experiences (e.g., the taste of sublingual buprenorphine, procedural difficulties and indignities of daily dosing, daily reminders of their addiction) experiencing fewer opioid withdrawal symptoms, having extra time for work and other activities, and reduced opportunities for MOUD diversion. Participants who viewed LAI-Bupe less favorably preferred to maintain their daily dosing routine; they liked daily time out of their housing unit, wanted to know what was “going into my body everyday”, and feared needles and adverse events. Participants also reported that jail-based clinicians used LAI-Bupe for patients who were previously caught diverting sublingual buprenorphine while incarcerated, suggesting limited patient participation in decision-making around LAI-Bupe initiation in some jails. This qualitative work identified barriers and facilitators to adoption of this important treatment modality, as well as jail-based policies and protocols that could be considered to broaden treatment options in jails that are considering implementation of LAI-Bupe treatment [12].

*Co-occurring mental health conditions*. We also identified the close interplay of substance use and mental health disorders in jails, demonstrating how MOUD implementation was perceived among jail staff to affect existing mental health and substance use services [13]. Provision of MOUD was perceived by jail staff to reduce acute mental health needs of individuals at intake by preventing withdrawal related symptoms, and thereby reducing suicide risk and behavioral instability. This effect of MOUD decreased immediate demands on mental health and correctional staff at intake but was reported to shift service demands to later parts of incarceration because individuals on MOUD received substance use counseling alongside medication. Counseling requirements, which differed by jail, appeared to be shaped by erroneous beliefs among staff [14], for example that counseling is clinically necessary in all cases or that counseling should be required to supplement MOUD. As more staff were needed to provide substance use counseling, an added challenge was to distinguish staff training needs and capabilities. Moreover, the delivery of substance use counseling to all individuals receiving MOUD presented logistical challenges, including privacy concerns, movement restrictions, and security requirements. Interestingly, we also learned that provision of MOUD helped to improve interdisciplinary collaboration, whereby mental health, medical, addiction, and corrections staff communicated more regularly around treatment planning and progress for individuals receiving MOUD.

### Outer context

*Legislative mandate.* Study participants, encompassing clinical jail staff, correctional officers, and jail administrators, acknowledged that the distinct policy context, in which the state legislature mandated almost half of all state jails to provide MOUD, facilitated implementation of the MOUD program [9]. At the same time, lawsuits and settlements with the Department of Justice drove other major changes in MOUD provision across other carceral-legal and community agencies (e.g., courts, community corrections, skilled nursing facilities).

Notably, the U.S. Attorney for Massachusetts issued a statewide compliance effort, as aligned with federal civil protections for uninterrupted MOUD access under the Americans with Disabilities Act, requiring all state and county correctional facilities to maintain MOUD treatment for individuals already prescribed them prior to custody. By April 2022, all remaining correctional facilities in Massachusetts provided access to all three forms of MOUD [15]. Together, this context created a system-wide environment in which MOUD adoption was perceived by jail leadership to be required. As a result, jails shifted from discussions of whether all types of MOUD should be offered to how implementation would occur in practice. Importantly, the legislative mandate included some financial support for key components of implementation, particularly around administrative requirements.

*Community-based MOUD providers*. Community-based MOUD providers were essential to both service delivery in the jail and in meeting the legislative requirements for continuity of treatment for incarcerated people returning to the community [16, 18]. Providers working with the jails described widespread staffing shortages and additional demands on existing staff. They emphasized a need to hire staff who were comfortable working with justice system-involved persons with co-occurring substance use and mental health conditions. To collaborate with jails, which are typically opaque and highly hierarchical organizations, community provider agencies had to be flexible and adapt quickly to the rapidly evolving needs of the jails. The fragmented nature of jail services was particularly challenging, and community providers often struggled to understand who was responsible for which service. As with jails, community agencies trained their own staff and provided education to jail staff about MOUD and community services, which strengthened interagency relationships.

### Bridging factors

*Re-entry planning*. MOUD pre-release policies and practices to plan for community re-entry were essential to providing opportunities for warm hand-offs to community-based MOUD treatment providers, with the goal of MOUD treatment continuity post-release. Jail staff involved with pre-release planning [17] and community-based treatment staff [18] identified barriers and facilitators to MOUD treatment continuity. Facilitators of treatment continuity included well-organized and thorough pre-release planning involving collaboration between pre-release planners and MOUD treatment providers, provision of last dose letters and bridge prescriptions, and transportation access. Barriers included no communication between jail and community-based providers, delays in insurance reinstatement, lack of cell phones and identification cards to facilitate acquisition of MOUD prescriptions at local pharmacies, and limited transportation options. Individuals who had received MOUD while incarcerated echoed these themes, emphasizing the importance of early and consistent reentry planning that encompasses social determinants of health, with a particular focus on transportation [19].

*Post-release risk environment*. Participants recognized the post-release environment as posing added health risks, with vulnerabilities heightened by patients’ reduced opioid tolerance and polysubstance use, toxicity of the regional drug supply, housing insecurity, limited employment opportunities, and limited availability of community-based MOUD treatment [19, 20]. Findings underscore the value of MOUD program components, such as provision of harm reduction supplies and low barrier access to community-based MOUD treatment, that enabled patients to navigate the post-release risk environment.

## MOUD treatment in jail improves treatment engagement, reduces recidivism and saves lives

A longitudinal treatment outcome study examined post-release MOUD initiation, engagement and retention, as well as fatal and non-fatal opioid overdose, and recidivism among incarcerated individuals with opioid use disorder (OUD) who received buprenorphine, methadone, naltrexone, or no MOUD in jail. Data were collected from seven jails on a total of 6,383 incarcerated people with OUD who were jailed between September 2019 and December 2020 and released before July 2021. Six months of post-release follow-up data were collected. Analyses compared the outcomes of those who did (*n* = 2,723 [43%]) and did not (*n* = 3,660 [57%]) receive MOUD treatment while incarcerated. As hypothesized, receipt of MOUD in jail was associated with an increased likelihood of post-release MOUD initiation and decreased risks of opioid overdose, reincarceration, and death from any cause [21]. These positive health outcomes underscore that provision of a MOUD program is a critical way that jails can reduce health and social harms, and thereby improve public health and public safety. Findings provide support for expansion of carceral MOUD programming nationwide.

## Understanding costs to inform MOUD program designs, budgetary needs, and resource allocation

We calculated the cost to the correctional system of providing MOUD in jail. This work began by identifying the resources necessary to implement and sustain the intervention at each site. A budget impact tool codified the necessary resources, and estimated associated costs [22]. Resources were valued using nationally-representative unit costs and categorized according to whether they were utilized as “fixed start-up” (incurred, e.g., accreditation fees and trainings), “time-dependent” (e.g., medication delivery and staff meetings), or “variable” (e.g., provider time and medication administered). Resource categories were then organized according to the relevant phase (i.e., implementation, sustainment phases). The budget impact tool is publicly available [23] and can be used by decision-makers to create facility-specific budgets and consider the trade-offs of different models.

This work identified three MOUD service delivery models [24]. Model 1 consisted of the jail contracting with a vendor to administer methadone daily, while clinical jail staff were responsible for administering buprenorphine and naltrexone. Model 2 involved facilities contracting with a certified vendor to run an in-house OTP and administer all three medications, while jails in Model 3 engaged in the complex regulatory process of obtaining their own internal OTP certifications and licenses. Implementation costs differed substantially across sites, due to various factors including changing models, dispensary construction, software development, and others. However, upon reaching the sustainment phase, monthly per-patient costs ranged from $93 to $242, inclusive of time-dependent, and variable costs, but excluding one-time fixed start-up costs already incurred during implementation.

## Conclusion

Our MassJCOIN study identified carceral MOUD treatment implementation policies, procedures, and experiences that can help to inform opioid use disorder treatment policies and legislation in other jurisdictions. We identified a broad array of considerations, best practices, and lessons learned about program implementation, which can help inform implementation elsewhere. Harm reduction principles reflected in findings thus far center on practices and processes that support MOUD treatment continuity such as orienting toward patient preference and shared decision-making, offering low barrier access to care, and supporting therapeutic versus punitive approaches, ultimately decreasing opioid-related morbidity and mortality outcomes. MassJCOIN continues to add to our knowledge base. Our work-in-progress assesses the comparative effectiveness of the three types of MOUD—methadone, buprenorphine, extended-release naltrexone—on post-release outcomes (Bovell-Ammon et al., unpublished observations), documents racial and ethnic disparities in MOUD receipt during incarceration and variation across facilities (Morgan et al., unpublished observations), and identifies implementation strategies to ensure life-saving treatment reaches all incarcerated individuals equitably (Gaba et al., unpublished observations). For county sheriffs who lead jails and houses of correction, and state officials who lead prisons, our study provides guidance for implementation and resource allocation, helping to inform policy change, which is greatly needed across many carceral settings. Program outcomes point to significant reductions in health and social harms among people who received MOUD treatment while incarcerated, including reductions in recidivism, overdose risk and all-cause mortality.

A broader implication is that the carceral MOUD program effectively enabled jails to be a part of solutions for addressing the opioid overdose epidemic rather than worsening it. Notably, prior to implementation of the carceral MOUD program most people with opioid use disorder were unable to receive MOUD treatment during incarceration and thus suffered excruciating opioid withdrawal symptoms and concomitant distress. Also, reductions in opioid tolerance during incarceration in the absence of MOUD put persons at excessive risk of overdose and death immediately post-release. In contrast, the carceral MOUD program underscored how jails and prisons can function as an important part of the community healthcare system. Jails were able to innovate to help incarcerated people with opioid use disorder meet health and social needs. Also, the MOUD program helped community agencies learn more about how to collaborate with jails to achieve common public health and safety goals. For these reasons, MOUD programs represent a way for carceral systems to work for the common good, enhance health and harm reduction policies, garner public trust and support, and increase the legitimacy and value of the carceral-legal system as a public institution.

At a national level, health policies focused on carceral health can be systematically improved. Few US jails offer MOUD treatment even when an incarcerated person with OUD requests it [25] and contrary to current guidance [26]. Advances in MOUD treatment policy in jails and prisons across the US can help to fill major gaps in prevention, treatment, and care, ultimately reducing healthcare costs when people who are incarcerated return to their home communities. While lessons learned are compelling, the unique legislative framework in Massachusetts may limit their transferability to other jurisdictions. Taken in its entirety, however, the MassJCOIN study provides a playbook for carceral settings that wish to implement MOUD treatment programs. As the value of harm reduction policies are considered in a wide range of settings across the US, MOUD treatment within jails and prisons can fill gaps in public health prevention and clinical care to reduce overdose and other concomitant risks.

## Data Availability

No datasets were generated or analysed during the current study.

## Abbreviations

EPIS: Exploration, preparation, implementation, sustainment
MassJCOIN: Massachusetts justice community opioid innovation network
MOUD: Medications for opioid use disorder
OUD: Opioid use disorder
